# Potent Anticancer Effects of Epidithiodiketopiperazine NT1721 in Cutaneous T Cell Lymphoma

**DOI:** 10.3390/cancers13133367

**Published:** 2021-07-05

**Authors:** Min Lin, Claudia M. Kowolik, Jun Xie, Sushma Yadav, Larry E. Overman, David A. Horne

**Affiliations:** 1 Department of Molecular Medicine, City of Hope National Medical Center, 1500 E. Duarte Road, Duarte, CA 91010, USA; mlin@coh.org (M.L.); jxie@coh.org (J.X.); syadav@coh.org (S.Y.); 2 Department of Translational Research and Cellular Therapeutics, City of Hope National Medical Center, 1500 E. Duarte Road, Duarte, CA 91010, USA; 3 Department of Chemistry, University of California, Irvine, CA 92697-2025, USA; leoverma@uci.edu

**Keywords:** cutaneous T cell lymphoma (CTCL), epidithiodiketopiperazine, NT1721, GLI1, STAT3

## Abstract

**Simple Summary:**

Cutaneous T cell lymphomas (CTCLs) are a group of blood cancers that cannot be cured with current chemotherapeutical or biological drugs. Patients with advanced disease are severely immunocompromised due to the unchecked expansion of malignant T cells and have low survival rates of less than four years. Hence, new treatment options for CTCLs are urgently needed. In this study the anti-CTCL activity of a new compound, NT1721, was determined in vitro and in two CTCL mouse models. We found that NT1721 increased apoptosis (programmed cell death) in the malignant T cells and reduced tumor growth better than two drugs that are currently clinically used for CTCL treatment (i.e., gemcitabine, romidepsin). These results suggest that NT1721 may represent a potent new agent for the treatment of advanced CTCL.

**Abstract:**

Cutaneous T cell lymphomas (CTCLs) are a heterogeneous group of debilitating, incurable malignancies. Mycosis fungoides (MF) and Sézary syndrome (SS) are the most common subtypes, accounting for ~65% of CTCL cases. Patients with advanced disease have a poor prognosis and low median survival rates of four years. CTCLs develop from malignant skin-homing CD4^+^ T cells that spread to lymph nodes, blood, bone marrow and viscera in advanced stages. Current treatments options for refractory or advanced CTCL, including chemotherapeutic and biological approaches, rarely lead to durable responses. The exact molecular mechanisms of CTCL pathology remain unclear despite numerous genomic and gene expression profile studies. However, apoptosis resistance is thought to play a major role in the accumulation of malignant T cells. Here we show that NT1721, a synthetic epidithiodiketopiperazine based on a natural product, reduced cell viability at nanomolar concentrations in CTCL cell lines, while largely sparing normal CD4^+^ cells. Treatment of CTCL cells with NT1721 reduced proliferation and potently induced apoptosis. NT1721 mediated the downregulation of GLI1 transcription factor, which was associated with decreased STAT3 activation and the reduced expression of downstream antiapoptotic proteins (BCL2 and BCL-xL). Importantly, NT1721, which is orally available, reduced tumor growth in two CTCL mouse models significantly better than two clinically used drugs (romidepsin, gemcitabine). Moreover, a combination of NT1721 with gemcitabine reduced the tumor growth significantly better than the single drugs. Taken together, these results suggest that NT1721 may be a promising new agent for the treatment of CTCLs.

## 1. Introduction

Cutaneous T cell lymphomas (CTCLs) are a biologically and clinically heterogeneous group of rare, incurable non-Hodgkin lymphomas that typically affect adults with a median age of 55 to 60 years [1,2,3]. The annual age-adjusted incidence rate in the United States is 6.4 per million persons [3]. CTCLs develop from malignant skin-homing CD4^+^ T cells that spread to lymph nodes, blood, bone marrow and viscera in advanced stages [4]. The most common subtypes, mycosis fungoides (MF) and Sézary syndrome (SS), account for 65% of CTCL cases [5,6]: MF is an indolent disease with only 10% of patients progressing to advanced stages, while SS represents the aggressive leukemic variant of CTCL that can develop de novo or progress from MF [7]. Differences in molecular profiles and responses to therapies suggest that MF and SS are distinct diseases [8,9]. Patients with SS present with generalized erythroderma and lymphadenopathy and have a poor prognosis with low median survival rates of 2–4 years [10]. SS is characterized by the clonal expansion of circulating malignant T cells with cerebriform nuclei (Sézary cells), which leads to the loss of a normal T cell receptor repertoire and thus immunosuppression [10,11,12,13,14]. Hence, patients with advanced disease are highly susceptible for opportunistic infections, which represent the most common CTCL-related cause of death [5,14]. Current treatment options for CTCL depend on the tumor stage. Unlike early stage CTCL, which is treated with skin-directed therapies, refractory early stage and advanced stage CTCL requires systemic mono- or combination therapy such as photopheresis, interferon alpha, bexarotene, monoclonal antibodies (e.g., mogamulizumab), histone deacetylase inhibitors (e.g., romidepsin (FK228)) and chemotherapeutics (e.g., gemcitabine) [4,15]. Response rates for most treatments vary between 30 and 50% and are rarely durable (7.5 to 22.4 months) [15,16]. Relapses are common and treatment for the aggressive forms remains palliative, not curative despite recent advances with chemotherapeutic and biological approaches [4]. Moreover, current therapies often lead to progressive drug resistance [17]. Thus, new treatment options for advanced CTCL are urgently needed.

Numerous genomic and gene expression profile studies have been performed over the past decade to discover genes that play a role in CTCL pathogenesis and to find potential new targets for therapy [11,14,18,19]. However, the molecular mechanisms of CTCL pathogenesis remain obscure as there is little overlap among the study results, which highlights the genetic heterogeneity of CTCL [11,19,20]. Genes with the most frequently altered expression profiles in CTCL are associated with the constitutive production of cytokines characteristic for T helper type 2 cells, resistance to growth inhibition via TGFβ and apoptosis induction [11]. Importantly, apoptosis resistance rather than increased proliferation is thought to be the cause for the accumulation of malignant T cells in the early stages of CTCL [21,22]. Aberrantly activated STAT3 has been identified as one important factor in CTCL progression and apoptosis resistance [18,23,24,25]. Activated STAT3 has been associated with increased expression of antiapoptotic proteins (BCL2, BCL-xL) and decreased overall and progression-free survival in CTCL [23,26].

In this study, the anti-CTCL efficacy of an epidithiodiketopiperazine, NT1721, was investigated. Epidithiodiketopiperazines (ETPs) are a broad class of fungal metabolites with potent antitumor activity in multiple solid and non-solid tumors. We have shown in a previous study that NT1721, an ETP chosen from a library of ETPs because of its potent antitumor activity against various solid and non-solid tumors, attenuated hedgehog/GLI signaling through downregulation of GLI transcription factors [27]. Aberrant hedgehog/GLI signaling promotes tumorigenesis, tumor progression, metastasis and drug resistance in various types of cancers [28,29]. Here we show that treatment with NT1721 mediated GLI1 downregulation in CTCL cells, which was associated with decreased STAT3 activation and reduced expression of downstream antiapoptotic proteins (BCL2 and BCL-xL). NT1721 led to potent apoptosis induction in vitro and significantly reduced tumor growth in two CTCL xenograft mouse models. Importantly, NT1721 impaired tumor growth significantly better than romidepsin or gemcitabine, two clinically used drugs for the treatment of refractory or relapsed CTCL [30,31]. Drug combinations of NT1721 with gemcitabine showed significantly improved antitumor effects in vitro and in vivo compared to the single agents. Our data suggest that NT1721 is, potentially, a promising new agent for the treatment of CTCL either by itself or in combination with gemcitabine.

## 2. Materials and Methods

### 2.1. Reagents

NT1721 was synthesized as previously described [32]. Gemcitabine, romidepsin (FK228) and the ERK inhibitor SCH772984 were purchased from Selleckchem (Houston, TX, USA).

### 2.2. Cell Culture

HUT78 and HH cells were obtained from ATCC (Manassas, VA, USA), authenticated by STR-profiling at the source and passaged for less than 6 months after receipt or resuscitation. Peripheral blood mononuclear cells (PBMCs) were obtained from ReachBio (Spokane, WA, USA). All cells were grown in RPMI supplemented with 10% FBS (Atlas Biologicals, Fort Collins, CO, USA). CD4^+^ cells were isolated from PBMCs using Dynabeads (ThermoFisher, Waltham, MA, USA).

### 2.3. Determination of IC_50_ Values

The CellTiter-GLO viability assay (Promega, Madison, WI, USA) was used according to the manufacturer’s instructions to determine cell viability: Briefly, 10,000 cells/well were seeded in 96-well plates, cultured overnight and then treated with various concentrations of NT1721 for 48 h or 72 h. Data from the assay were expressed as percent of viable cells compared to the vehicle control (0.1% DMSO).

### 2.4. Proliferation Assay

Cells were stained with CFSE ((5(6)-carboxyfluorescein N-hydroxysuccinimidylester, CellTrace, ThermoFisher) according to the manufacturer’s instructions, and seeded at a concentration of 10,000 cells/well. The cells were treated with NT1721 or DMSO the next day, harvested after 48 h or 72 h, stained with 0.2 μg/mL DAPI and subjected to FACS analysis. Fluorescence data were collected on a CyAN flow cytometer (Beckman Coulter, Brea, CA, USA) and analyzed with FlowJo software (TreeStar, Ashland, OR, USA).

### 2.5. Cell Cycle Analysis

Cells were treated with NT1721 as indicated and harvested after 48 h, stained with propidium iodide (PI) (ThermoFisher) as previously described and subjected to FACS analysis [27].

### 2.6. QPCR

Total RNA was isolated using the Direct-zol kit (Zymo Research, Irvine, CA, USA) and reverse transcribed using the Tetro cDNA synthesis kit (Bioline, Taunton, MA, USA). The following qPCR primers were used:

BCL2: 5′AGTACCTGAACCGGCACCT/5′GCCGTACAGTTCCACAAAGG

BCL-xL: 5′CTGAGTTACCGGCATCCC/5′TTCTGAAGGGAGAGAAAGAGATTC

BMI1: 5′CCATTGAATTCTTTGACCAGAA/5′CTGCTGGGCATCGTAAGTATC

CCNE1: 5′GGCCAAAATCGACAGGAC/5′GGGTCTGCACAGACTGCAT;

GAPDH: 5′AGCCACATCGCTCAGACAC/5′GCCCAATACGACCAAATCC;

GLI1: 5′ACCCGGGGTCTCAAACTG/5′GGCTGACAGTATAGGCAGAGC;

Quantitative PCR was performed using a CFX96 Touch Real-Time PCR detection system (Bio-Rad, Hercules, CA, USA). Relative expression levels were calculated using the 2^−ΔΔCt^ method and GAPDH as reference gene.

### 2.7. Western Blots

SDS-PAGE and Western blots were carried out as previously described [27]. Briefly, cells were lyzed using RIPA buffer (Cell signaling, Danvers, MA, USA) supplemented with Halt Protease inhibitor cocktail (ThermoFisher). The protein concentration was quantified using the Pierce BCA Protein Assay Kit (ThermoFisher). Equal amounts of protein were loaded on precast gels (Bio-Rad, Hercules, CA, USA) and transferred to PVDF membranes (Bio-Rad) using the Trans-Blot Turbo Transfer System (Bio-Rad). Membranes were blocked for 1 h at RT in blocking buffer (5% *w*/*v* nonfat dry milk, 0.1% Tween-20 in TBS), then incubated with primary antibodies overnight at 4 °C. The NE-PER Nuclear and Cytoplasmic Extraction Kit (ThermoFisher) was used for the preparation of cytoplasmic and nuclear extracts. Primary antibodies were purchased from Cell Signaling Technology (Danvers, MA, USA): β-actin (#4970), BCL2 (#4223), BCL-xL (#2764), BMI1 (#6964), cleaved PARP (#5625), cleaved CASP3 (#9664), ERK (#4695), pERK1/2 (#4370), GAPDH (#5174), GLI1 (#3538), H2AX (#2595), γH2AX (#9718), H3 (#4499), p21 (#2947), p27 (#3686), STAT3 (#9139), pSTAT3 (Y705) (#9145), TBP (#44059); additionally, tubulin (05-829) was purchased from Sigma (St. Louis, MO, USA). The membranes were washed three times with TBS and then incubated 1 h at RT with the appropriate secondary antibodies. Bands were visualized using X-ray film or the ChemiDoc Imaging System (Bio-Rad).

### 2.8. In Vivo Studies

Mouse care and experimental procedures were performed under pathogen-free conditions in accordance with approved protocols from the institutional animal care and use committee of City of Hope National Medical Center. For the xenograft mouse models, 5 million HuT78 or HH cells were injected subcutaneously into the flank of 6- to 8-week old NSG mice (Jackson Laboratory, Bar Harbor, ME, USA). The tumor volume was calculated using the equation: V = ½ × W^2^ × L with W and L representing the width and length. Treatment started when the tumor volume reached ~200 mm^3^. The mice were then distributed into groups bearing equal tumor burdens and treated with NT1721 by gavage, gemcitabine (i.p. injection), romidepsin (i.p. injection) or the vehicle control as indicated. PBS was used as vehicle control for treatments by i.p. injections; 30% solutol/3.3% DMSO in PBS or Ora-Blend (ThermoFisher)/10% saline were used as vehicle controls for gavage treatments.

### 2.9. Statistical Analysis

The mean ± standard deviation (SD) was calculated for each treatment group. The two-tailed *t*-test (GraphPad Software Inc., La Jolla, CA, USA) was used to determine statistical significance between two treatment groups in the in vitro studies. The Mann–Whitney U test was used to determine statistical significance between treatment groups in mouse models. *P* values < 0.05 were considered significant.

## 3. Results

### 3.1. NT1721 Led to Reduced CTCL Cell Viability, Proliferation and G2 Cell Cycle Arrest at Nanomolar Concentrations

**Figure 1:**
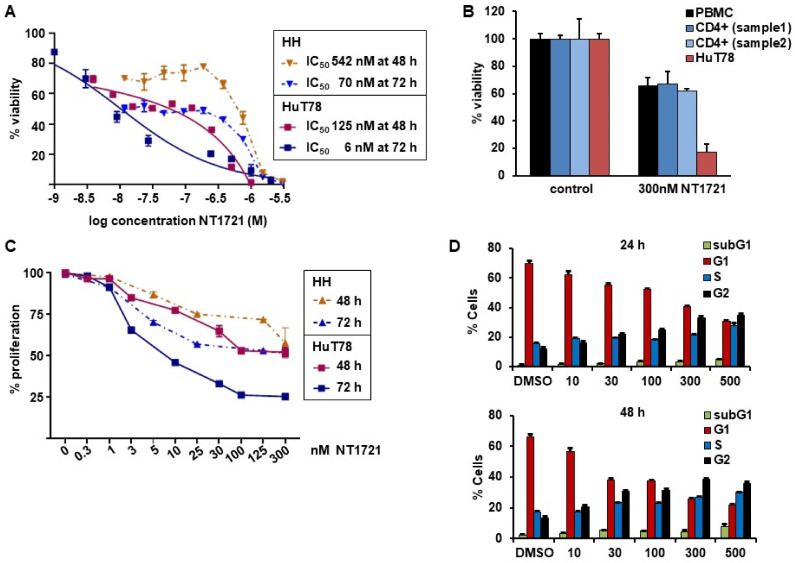
NT1721 decreased CTCL cell viability, proliferation and led to G2 cell cycle arrest. (**A**) Cell viability. HuT78 and HH cells were treated with increasing concentrations of NT1721. Cell viability and IC_50_ values were determined after 48 and 72 h. (**B**) Effect of NT1721 on normal cells. HuT78 cells, normal PBMCs and normal CD4^+^ cells were treated with 300 nM NT1721 or 0.3% DMSO. (**C**) Proliferation. CTCL cells were stained with CFSE, treated with NT1721 and subjected to FACS analysis after 48 or 72 h to determine the mean fluorescence intensity. The data were normalized to the controls. (**D**) Cell cycle analysis. HuT78 cells were treated with NT1721, stained with PI after 24 h and 48 h and analyzed by FACS. The graphs represent the mean ± SD from triplicate values.

To assess the potency of NT1721 against CTCL, we treated HuT78 and HH cells with increasing concentrations of NT1721 and determined viability after 48 and 72 h. The IC_50_ values were in the nanomolar range for both cell lines and time points: 125 nM and 6 nM in HuT78 cells after 48 and 72 h, respectively; 542 nM and 70 nM in HH cells after 48 and 72 h, respectively (Figure 1A). To assess the effect of NT1721 on normal cells we also treated Hut78 cells, normal peripheral blood mononuclear cells (PBMCs) and CD4+ cells from two healthy donors with NT1721. As shown in Figure 1B, 300 nM NT1721 reduced the viability of HuT78 by 83%, while only decreasing the viability of normal PBMCs and normal CD4^+^ by ~32%. The result suggests that NT1721 preferentially decreased the viability of CTCL cells. To investigate the effect of NT1721 on CTCL proliferation we stained HuT78 and HH cells with ((5(6)-carboxyfluorescein N-hydroxysuccinimidylester (CFSE) and treated them with NT1721 (up to 300 nM) for 48 or 72 h. FACS analysis of live cells showed that NT1721 reduced proliferation faster and at lower concentrations in Hut78 compared to HH cells (Figure 1C). However, treatment with 100 nM NT1721 dramatically reduced proliferation in both cell lines after 72 h by 50–75%.

We then determined the effect of NT1721 on the cell cycle by staining NT1721-treated cells with PI. As shown in Figure 1D, NT1721 led to cell cycle arrest in CTCL cells. Treatment with NT1721 led to G2 cell cycle arrest as indicated by the dose- and time-dependent increase in the percentage of cells in the G2 phase and concomitant decrease in the percentage of cells in the G1 phase. Treatment with 500 nM NT1721 decreased the number of cells in the G1 phase from ~70% to 30% and 22% after 24 h and 48 h, respectively; the number of cells in the G2 phase increased from ~12% to 35 and 38% in NT1721-treated cells after 24 h and 48 h, respectively. Taken together, the results indicate that NT1721-mediated preferential reduction in CTCL cell viability (compared to normal CD4^+^ cells) was associated with decreased proliferation and G2 cell cycle arrest.

### 3.2. NT1721 Downregulated GLI1 Transcription Factor in CTCL Cells

Since we previously found that NT1721 potently downregulated GLI transcription factors [27], we first determined the effect of NT1721 on GLI1 expression in CTCL cells.

**Figure 2:**
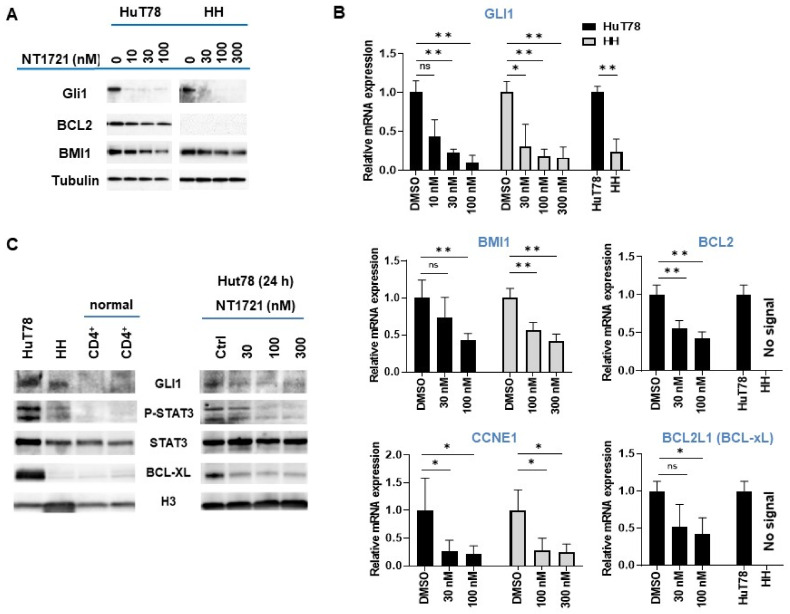
NT1721 downregulated GLI and GLI target genes in CTCL cells. (**A**) Protein expression levels were assessed by Western blot after 24 h treatment with NT1721. (**B**) QPCR analysis of gene expression. The data were analyzed using GAPDH as the reference gene and represent the mean ± SD from 3 independent experiments. (* indicates *p* ≤ 0.05; ** indicates *p* ≤ 0.01) (**C**) GLI1, pSTAT3, STAT3 and BCL-xL protein expression in normal CD4^+^ cells, CTCL cells and NT1721-treated CTCL cells. Original Western Blots of Figure 2A,C available in Figure S4.

As shown in (Figure 2A,B), NT1721 dramatically reduced GLI1 expression on the mRNA and protein level in both HuT78 and HH cells after 24 h at concentrations of ≤100 nM. A comparison of GLI1 expression levels in the CTCL cell lines revealed ~3-fold higher GLI1 mRNA expression in Hut78 as well as higher GLI1 protein expression compared to HH cells (Figure 2B,C). In contrast, no GLI1 protein expression was detected in two samples of normal CD4^+^ cells (Figure 2C), suggesting that GLI1 might be aberrantly expressed in the CTCL cell lines. Moreover, this result is in agreement with a previous report showing strong GLI1 expression in T cell lymphomas including CTCL while no GLI1 expression was observed in healthy peripheral blood T cells [33].

We next investigated the effect of GLI1 downregulation on the expression of GLI target genes that are involved in apoptosis resistance and cell cycle progression (i.e., BCL2, BMI1, CCNE1) and that were also downregulated by NT1721 in our previous study [27]: NT1721 concentrations of 100 nM significantly reduced BMI1 and CCNE1 expression in HuT78 and HH cells (Figure 2A,B). NT1721 also significantly decreased BCL2 expression in HuT78 (Figure 2A,B). However, in line with previous reports [17,34], no BCL2 mRNA or protein expression could be detected in HH cells.

We decided to determine the effect of NT1721 on STAT3 activation since several reports show that GLI1 inhibition or knockdown decreased phosho-STAT3 (pSTAT3) levels in T cell lymphomas and that pSTAT3 may be an important driver of CTCL, especially in advanced disease [26]. As shown in Figure 2C, strong pSTAT3 was only detected in the CTCL cells and not in the normal CD4^+^ samples. Treatment of CTCL cells with NT1721 resulted in reduced pSTAT3 levels (Figure 2C). The reduced STAT3 activation was accompanied by a significant reduction in mRNA and protein levels of the STAT3 target gene BCL-xL in Hut78 (Figure 2B,C). In contrast, very low BCL-xL expression was detected in HH and normal CD4^+^ cells.

### 3.3. NT1721 Induced Apoptosis in CTCL In Vitro and In Vivo

**Figure 3:**
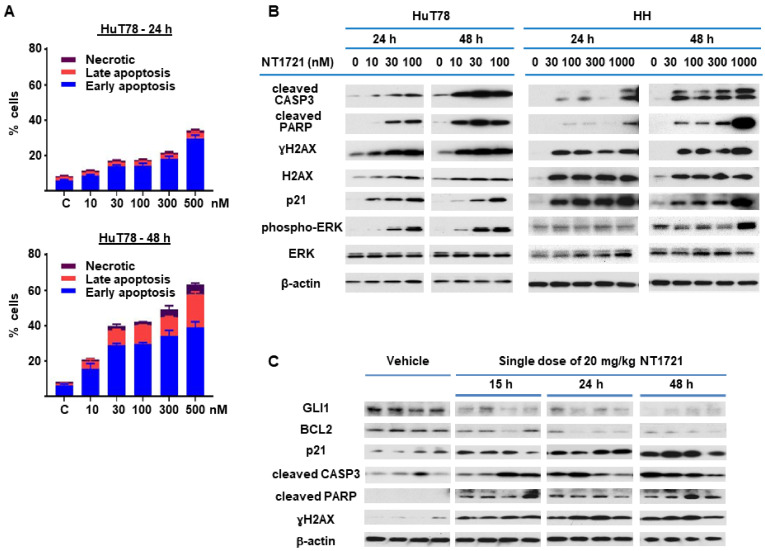
NT1721 induced apoptosis in CTCL cells in vitro and in vivo. (**A**) HuT78 cells were treated with NT1721 for 24 h or 48 h, then stained with annexin V and subjected to FACS analysis. (**B**) HuT78 and HH cells were treated with NT1721 as indicated. Western blots were used to analyze the expression of genes related to apoptosis induction. (**C**) Apoptosis induction in vivo. NSG mice harboring HuT78 tumors (*n* = 4 per group) were treated by gavage with a single dose of NT1721 (20 mg/kg) or the vehicle (3.3% DMSO/30% solutol in PBS). The tumors were then harvested at the indicated time points and subjected to Western blot analysis. Original Western Blots of Figure 3B,C available in Figure S5.

Since NT1721 mediated the downregulation of antiapoptotic BCL2 and BCL-xL, we decided to further investigate the effect of NT1721 on apoptosis. HuT78 cells were treated with NT1721 (up to 500 nM) and stained with annexin V after 24 h and 48 h. As shown in Figure 3A, low concentrations of NT1721 (10–30 nM) significantly increased the percentage of apoptotic cells to 20–40% after 48 h. Treatment with a higher dose of NT1721 (500 nM) resulted in 35% and 62% apoptotic cells after 24 h and 48 h, respectively. To further investigate apoptosis induction by NT1721 on the molecular level, NT1721-treated HuT78 and HH samples were analyzed by Western blots. As shown in Figure 3B, NT1721 increased the expression of proteins related to apoptosis induction (i.e., cleaved CASP3 and cleaved PARP) in a concentration- and time-dependent manner. Moreover, this increase in apoptotic markers was accompanied by H2AX upregulation and increased levels of γ H2AX. Subcellular fractionation revealed that in NT1721-treated Hut78 cells the cytoplasmic fractions contained the majority of γH2AX and total H2AX; only a small amount of these proteins was detected in the nuclear fractions (Figure S1). Also observed was a dramatic increase in γH2AX and total H2AX levels in NT1721-treated HH cells; a significant portion of these proteins was also found in the cytoplasmic fractions. This H2AX upregulation and cytoplasmic localization provided additional support that NT1721 induced apoptosis in CTCL. Increased H2AX levels have been identified as a cause of apoptosis in previous studies [35,36].

Since ERK activation has been linked to both apoptosis induction and G2 cell cycle arrest [37,38], the effect of NT1721 on ERK activation and p21 (CDKN1A) levels was investigated. As shown in Figure 3B, low concentrations of NT1721 (10–30 nM) led to concentration-dependent activation of ERK in Hut78 starting after 24 h. In HH cells, ERK activation was detected after 48 h of treatment with NT1721 and at higher NT1721 concentrations. Dramatic upregulation of p21 by NT1721 was detected in both cell lines (Figure 3B). To explore the importance of ERK phosphorylation on apoptosis induction, HuT78 cells were treated with an ERK inhibitor SCH772984, NT1721, or a combination of both agents. Western blot analyses showed that NT1721 alone increased phospho-ERK (pERK), p21, cleaved CASP3, cleaved PARP and γH2AX levels in a concentration-dependent manner (Figure S2A). In contrast, SCH772984 alone had no effect on these protein levels. Samples treated with combinations of NT1721 and SCH772984 displayed reduced ERK phosphorylation, cleaved PARP, cleaved CASP3, p21 and γH2AX induction compared to samples treated with NT1721 alone. To further investigate the effect of the drug combination on apoptosis induction we stained Hut78 cells that were treated with the drug combination with annexin V. As shown in Figure S2B, treatment with both NT1721 and SCH772984 reduced the percentage of apoptotic HuT78 cells significantly compared to cells treated with NT1721 alone. Taken together, these results suggest that increased ERK phosphorylation may be critical for efficient apoptosis induction by NT1721. To evaluate whether treatment with NT1721 could also induce apoptosis in vivo, NSG mice were injected with HuT78 cells subcutaneously. When the tumor volumes reached ~200 mm^3^ the mice were treated with a single oral dose of NT1721 or the vehicle control. The mice were euthanized 15, 24, 48 or 72 h later, and tumor tissue from individual mice was used for Western blot analysis. As shown in Figure 3C, NT1721 increased cleaved CASP3, cleaved PARP and p21 expression while decreasing GLI1 and BCL2 expression, thus recapitulating the changes in protein expression that were observed in vitro. These results suggest that NT1721 induces apoptosis in CTCL in vivo.

### 3.4. NT1721 Suppressed Tumor Growth Significantly Better Than Romidepsin in CTCL Mouse Models

**Figure 4:**
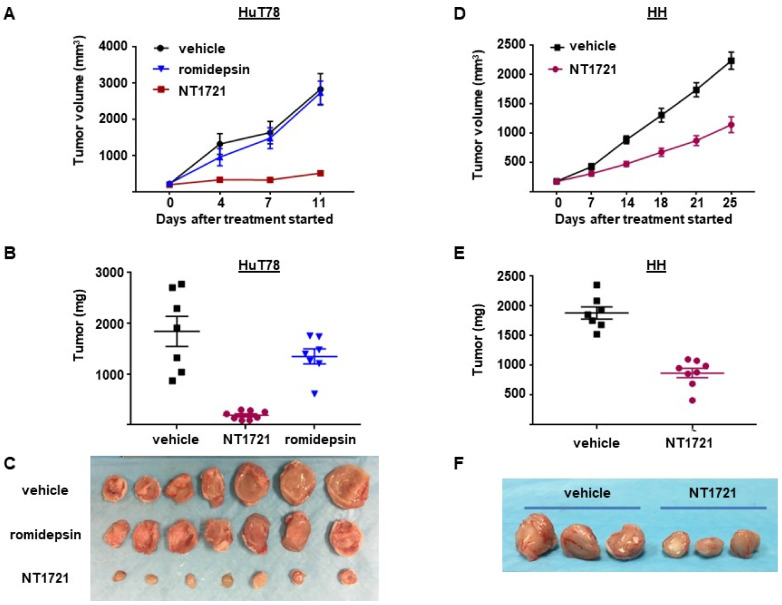
NT1721 suppressed tumor growth significantly better than romidepsin in a CTCL mouse model. (**A**) NSG mice (*n* = 7/group) were injected s.c. with HuT78 cells and treated by gavage three times/week (on consecutive days) with the vehicle control (30% solutol/3.3% DMSO in PBS), 20 mg/kg NT1721 or with 2 mg/kg romidepsin by I.P. injection twice per week. Treatment with NT1721 reduced tumor volumes significantly better than romidepsin on Day 4, 7 and 11 (*p* values < 0.01). (**B**) Weight of HuT78 tumors from individual mice (*p* = 0.0003 for control vs. NT1721 and for romidepsin vs. NT1721; no significant difference for control vs. romidepsin: *p* = 0.317). (**C**) Picture of HuT78 tumors from the treated mice. (**D**) NSG mice were injected s.c. with HH cells and treated by gavage on three consecutive days per week with 20 mg/kg NT1721 or the vehicle control. The differences in tumor volume were significant from day 14 through day 25 (*p* values < 0.0012). (**E**) Weight of HH tumors from individual mice (*p* = 0.0003). (**F**) Picture of representative HH tumors from the treated mice.

To assess the antitumor efficacy of NT1721 in vivo, NSG mice were injected subcutaneously with HuT78 cells. When the tumor volumes reached ~200 mm^3^ the mice were treated with 20 mg/kg NT1721 by gavage three times per week on consecutive days. The control group received the vehicle control (30% solutol/3.3% DMSO in PBS). As shown in Figure 4A,B, NT1721 reduced the tumor volume and tumor weight significantly by 90%. To compare the efficacy of NT1721 to romidepsin, a clinically used drug for CTCL treatment [39], mice were treated with romidepsin (2 mg/kg) twice per week. Romidepsin did not significantly reduce the tumor weight (Figure 4A,B). Moreover, romidepsin-treated mice showed signs of toxicity, i.e., ascites and diarrhea, while no obvious signs of toxicity were observed in NT1721-treated mice. We next investigated the in vivo efficacy of NT1721 against CTCL in another mouse model. NT1721 also reduced the tumor volume and tumor weight of HH tumors by ~50% (Figure 4D–F). Treatment of HH tumors with romidepsin did not result in smaller tumor volumes (data not shown). These results indicate that NT1721 reduced CTCL tumor growth better than romidepsin and that NT1721 may be less toxic than romidepsin.

### 3.5. DMSO-Free Formulation of NT1721 Potently Decreased CTCL Tumor Growth

**Figure 5:**
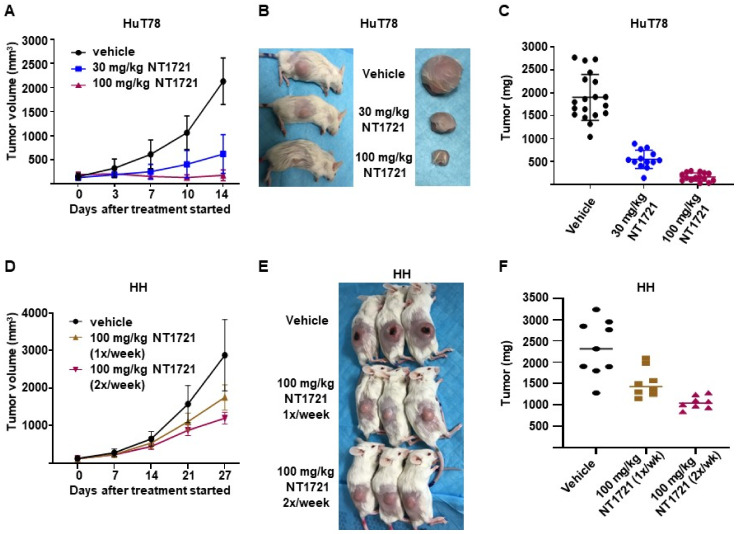
In vivo efficacy of a DMSO-free formulation of NT1721. (**A**–**C**) NSG mice bearing HuT78 tumors were treated with 30 mg/kg (five times per week), 100 mg/kg NT1721 (twice per week) or the vehicle control (Ora-Blend containing 10% saline). (**A**) Tumor volume. (**B**) Representative mice and tumors from the treatment and control groups. (**C**) Tumor weight in treated and control mice. The differences between control and treatment groups as well as between the treatment groups were statistically significant (*p* < 0.0001). (**D**–**F**) NSG mice bearing HH tumors were treated with 100 mg/kg NT1721 once a week, 100 mg/kg NT1721 twice per week or the vehicle control (Ora-Blend containing 10% saline). (**D**) Tumor volume. (**E**) Representative mice from the treatment and control groups. (**F**) Tumor weight in treated and control mice. The differences between control and treatment groups were statistically significant (control compared to 100 mg/kg 1×/week: *p* = 0.021 and control compared to 100 mg/kg 2×/week: *p* = 0.0002).

To assess the efficacy of NT1721 under more clinically relevant conditions we used a DMSO-free formulation of NT1721 for the treatment of mice bearing CTCL tumors: NT1721 was suspended in Ora-Blend containing 10% saline. Mice bearing HuT78 tumors were treated with the vehicle control, 30 mg/kg five times per week or 100 mg/kg NT1721 twice per week. As shown in Figure 5A–C, treatment with 30 mg/kg or 100 mg/kg NT1721 reduced the tumor volume and tumor weight significantly by 70% and 91%, respectively (*p* < 0.0001). No weight loss or other obvious signs of toxicity were observed with this formulation and drug schedule. In addition, mice bearing HH tumors were treated once or twice per week with 100 mg/kg NT1721 (in Ora-Blend). As shown in Figure 5D–F, the treatment led to a significant reduction in tumor growth by 35% and 55%, respectively. Taken together, the results show that treatment with NT1721 administered in a DMSO-free, more clinically relevant formulation potently suppressed CTCL tumor growth in mice.

### 3.6. In Vivo Combinations of NT1721 and Gemcitabine Suppressed CTCL Tumor Growth Better Than the Single Agents

**Figure 6:**
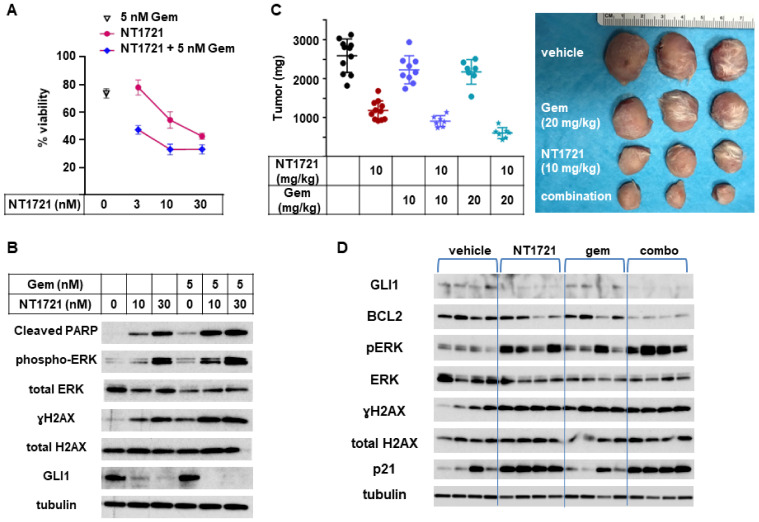
In vivo combination of NT1721 with gemcitabine. (**A**) Cell viability of HuT78 cells treated with NT1721, gemcitabine or a combination of the two drugs. (**B**) Protein expression of GLI1 and proteins related to apoptosis iHut78 H. Induction (cleaved PARP, pERK, γH2A.x) in HuT78 cells treated with NT1721, gemcitabine or a combination of the two drugs for 48 h. (**C**) Tumor weight in mice treated with NT1721, gemcitabine, a combination of the two drugs with the vehicle controls (Ora-Blend containing 10% saline or PBS, respectively) for 3 weeks. Representative HuT78 tumors treated with the single drugs or drug combinations are shown on the right side. (**D**) Expression of GLI1 and proteins related to apoptosis iHut78 H. Induction (BCL2, p21, pERK, γH2AX) in individual mice treated with 10 mg/kg NT1721, 20 mg/kg gemcitabine or the drug combination for 3 weeks. Original Western Blots of Figure 6B,D available in Figure S6.

To determine if combinations of NT1721 with gemcitabine, a clinically used drug for CTCL treatment [39], could lead to improved anticancer responses, an in vitro viability assay was employed. As shown in Figure 6A, a combination of NT1721 with gemcitabine decreased the cell viability of HuT78 cells significantly better than the single drugs alone.

To investigate what might cause this effect we assessed the expression of proteins related to apoptosis induction as well as GLI1 in HuT78 samples that were treated with NT1721, gemcitabine, or the drug combination. As shown in Figure 6B, the expression of cleaved PARP, pERK, and γH2AX was increased in samples treated with the drug combination compared to samples treated with the single agents. This result suggests that the drug combination may increase apoptosis induction and hence decrease cell viability. Treatment with the drug combination also seemed to slightly enhance GLI1 downregulation while treatment with gemcitabine alone did not affect GLI1 expression.

To evaluate the efficacy of NT1721, gemcitabine, and the drug combination in vivo, mice bearing HuT78 tumors were treated with 10 mg/kg NT1721 (in Ora-Blend, twice per week), 10 mg/kg gemcitabine (twice per week), 20 mg/kg gemcitabine (twice per week) or the respective combinations. A comparison of the in vivo efficacy of NT1721 and gemcitabine showed that NT1721 significantly decreased tumor growth by 55%. Both 10 and 20 mg/kg gemcitabine only non-significantly decreased tumor growth by ~15% (Figure 6C). Better suppression of tumor growth by NT1721 was also observed in the other CTCL mouse model (HH tumors). As shown in Figure S3, NT1721 (100 mg/kg, 1×/week) decreased HH tumor growth by 52%, while gemcitabine (100 mg/kg, 2×/week) led to a 45% reduction. However, the difference between the two drugs did not reach statistical significance in this tumor model. Taken together, these results show that NT1721 led to a better suppression of CTCL tumor growth at a lower dose compared to gemcitabine.

Next, efficacies of the drug combinations (10 mg/kg NT1721 plus either 10 mg/kg or 20 mg/kg gemcitabine) against Hut78 tumors were compared. Mice treated with the drug combinations displayed 65% and 76% smaller tumors, respectively, while NT1721 alone only led to a 45% reduction (Figure 6C). The differences were significant: *p* = 0.0012 for NT1721 vs. NT1721 plus 10 mg/kg gemcitabine; *p* = 0.0003 for NT1721 vs. NT1721 plus 20 mg/kg gemcitabine. Thus, these results indicate that the drug combinations reduced tumor growth significantly better than the single drugs alone.

We then determined the expression of proteins related to the hedgehog pathway (GLI1, BCL2) and to apoptosis induction in individual mice treated with 10 mg/kg NT1721, 20 mg/kg gemcitabine or the drug combination. As shown in Figure 6D, NT1721 decreased the expression of GLI1 and antiapoptotic BCL2 and increased the expression of apoptosis induction-related proteins (p21, pERK, γH2AX), thus recapitulating what was observed in vitro (Figure 3B and Figure 6B). BCL2 expression appeared to be decreased to a greater extend in mice treated with the drug combination compared to mice treated with the single agents. Upregulation of pERK, which is essential for NT1721-mediated increased expression of p21, yH2AX, cleaved PARP and cleaved CASP3 (Figure S2B), was elevated to a greater extent in mice treated with the drug combination compared to mice treated with NT1721 alone (Figure 6D). Taken together these results indicate that combining gemcitabine with NT1721 resulted in better antitumor effects compared to treatment with the single agents. This improved anticancer effect appears to be mediated by increased apoptosis.

## 4. Discussion

Apoptosis resistance has been identified as a major cause for the accumulation of malignant T cells in CTCL [22]. Patients with advanced disease have a poor prognosis with low survivals rates of less than 4 years [40]. Since current treatment options for advanced CTCL stages are rarely durable and remain largely palliative [4], new strategies for the treatment of advanced CTCL are urgently needed.

Here we investigated the anti-CTCL activity of an ETP, NT1721. Treatment of CTCL cells derived from the blood of patients with either Sézary syndrome (Hut78) or aggressive, extensively pretreated CTCL (HH) (ATCC.org) with NT1721 downregulated GLI1 transcription factor, which was associated with decreased viability, reduced proliferation and increased apoptosis. This result aligns with previous reports showing that inhibition of aberrantly activated hedgehog/GLI in various types of T cell lymphomas by hedgehog inhibitors or GLI1 knockdown led to increased apoptosis and reduced proliferation and viability [33,41,42,43]. Decreased pSTAT3 levels as well as reduced expression of downstream targets were also observed in NT1721-treated cells. This agrees with a previous study showing that GLI1 knockdown or inhibition leads to a reduction in pSTAT3 levels in T cell lymphomas including CTCL [33]. Aberrant STAT3 activation plays a major role in CTCL since it has been linked to apoptosis resistance, progression, and inferior clinical outcome [23,24,25]. Both GLI1 expression and STAT3 activation are absent from healthy peripheral T cells according to previous reports [24,33,43,44] and our results. This might partially explain why NT1721 preferentially reduces the viability of CTCL cells. The NT1721-mediated decreased GLI1 expression and STAT3 activation led to concentration-dependent downregulation of downstream antiapoptotic proteins, i.e., BCL2 and BCL-xL, which contributed to apoptosis induction. Several recent studies have highlighted the importance of BCL2/BCL-xL inhibition for efficient apoptosis induction in CTCL. Adding BCL2 or BCL2/BCL-xL inhibitors to the treatment of CTCL cells with other agents (e.g., histone deacetylase or bromodomain inhibitors) synergistically increased apoptosis induction [7,17,34,45,46]. In contrast, no synergy was observed when BCL2-negative HH cells were treated with the drug combinations [17,34]. Moreover, it has been shown that antiapoptotic BCL2 proteins have a role in promoting hedgehog/GLI signaling by enhancing the turnover of a GLI antagonist, SUFU tumor suppressor. This inhibits GLI-SUFU interaction and thus increases the expression of GLI target genes such as BCL2 itself [47].

Our in vivo results suggested that orally available, well-tolerated NT1721 inhibits CTCL tumor growth significantly better than two clinically used drugs, i.e., romidepsin or gemcitabine, in two CTCL mouse models. However, NT1721 reduced tumor growth in mice bearing Hut78 tumors significantly better than in mice bearing HH tumors (85% and 60% inhibition, respectively). Lower GLI1 and pSTAT3 levels as well as the lack of BCL2/BLC-xl expression in HH cells might at least partially explain the lower in vitro sensitivity and relatively lower in vivo response to NT1721 compared to Hut78 cells. In summary, NT1721 may be a promising new agent for the treatment of CTCLs overexpressing GLI1 and activated STAT3 either as a single agent or in combination with gemcitabine.

## 5. Conclusions

NT1721 potently induces apoptosis in CTCL in vitro and in vivo. Orally available, well-tolerated NT1721 reduces CTCL tumor growth better than two drugs that are currently clinically used for CTCL treatment (romidepsin and gemcitabine). Moreover, combinations of NT1721 with gemcitabine suppress CTCL growth better than the single agents. Thus, our results provide a rationale for testing the anti-CTCL efficacy of NT1721 in the clinic either as a single agent or in combination with gemcitabine.

## 6. Patents

A patent related to this work was filed by City of Hope.

## Data Availability

The data presented in this study are available in this article.

## Supplementary Materials

The following are available online at https://www.mdpi.com/2072-6694/13/13/3367/s1. Figure S1: γH2AX expression. CTCL cells were treated with NT1721 as indicated for 24 h. γH2AX expression was detected in the cytoplasmic and nuclear extracts. Tubulin and TBP expression were used as loading controls; Figure S2: Combining NT1721 with an ERK inhibitor reduced apoptosis induction in HuT78 cells. (**A**) Protein expression in HuT78 cells treated with NT1721, the ERK inhibitor (SCH772984) or a combination. (**B**) Annexin V staining of HuT78 cells treated with NT1721, SCH772984 or combinations of both agents; Figure S3: In vivo efficacies of NT171 (in DMSO-free formulation) and gemcitabine against HH tumors. The mice were treated with NT1721, gemcitabine or the vehicle controls as indicated. (**A**) Tumor volume. (**B**) Tumor weight. (**C**) Picture of representative tumors from treatment and control groups; Figure S4: Original Western Blots of Figure 2A,C; Figure S5: Original Western Blots of Figure 3B,C; Figure S6: Original Western Blots of Figure 6B,D.
