# Correction: Lin et al. Potent Anticancer Effects of Epidithiodiketopiperazine NT1721 in Cutaneous T Cell Lymphoma. *Cancers* 2021, *13*, 3367

**DOI:** 10.3390/cancers13246151

**Published:** 2021-12-07

**Authors:** Min Lin, Claudia M. Kowolik, Jun Xie, Sushma Yadav, Larry E. Overman, David A. Horne

**Affiliations:** 1 Department of Molecular Medicine, City of Hope National Medical Center, 1500 E. Duarte Road, Duarte, CA 91010, USA; mlin@coh.org (M.L.); jxie@coh.org (J.X.); syadav@coh.org (S.Y.); 2 Department of Translational Research and Cellular Therapeutics, City of Hope National Medical Center, 1500 E. Duarte Road, Duarte, CA 91010, USA; 3 Department of Chemistry, University of California, Irvine, CA 92697-2025, USA; leoverma@uci.edu

There is an omission in the Institutional Review Board Statement and Conflict of Interest statements of the paper [1]. The authors would like to update the following parts: 

**Institutional Review Board Statement:** Mouse care and experimental procedures were performed under pathogen-free conditions in accordance with approved protocols from the institutional animal care and use committee of the City of Hope National Medical Center (City of Hope IACUC protocol # is 13053 approved 2 November 2014).

**Conflicts of Interest:** D.A.H. and L.E.O. are cofounders of Novonco Therapeutics Inc., Los Angeles, CA, USA. The other authors declare no conflict of interest. The funders had no role in the design of the study; in the collection, analysis, or interpretation of data; in the writing of the manuscript; or in the decision to publish the results.

We apologize for this error and state that the scientific conclusions are unaffected. The original article has been updated.
