# Supplementary material for: Potent Anticancer Effects of Epidithiodiketopiperazine NT1721 in Cutaneous T Cell Lymphoma
Source: Cancers (Basel). 2021 Jul 5;13(13):3367. doi: 10.3390/cancers13133367 (PMC8268131; doi:10.3390/cancers13133367)
Supplement: Supplementary file 1 [file cancers-13-03367-s001.zip › cancers-1244811-supplementary.pdf]

Supplementary Material: Potent Anticancer Effects of Epidithiodiketopiperazine NT1721 in Cutaneous T Cell Lymphoma

Min Lin, Claudia M. Kowolik, Jun Xie, Sushma Yadav, Larry E. Overman and David A. Horne

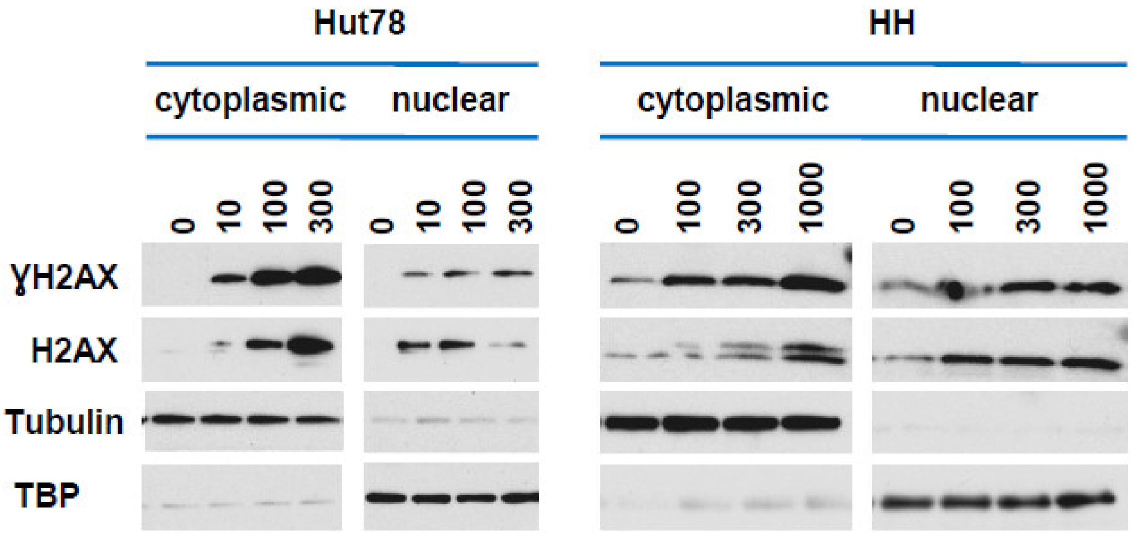

Figure S1. γH2AX expression. CTCL cells were treated with NT1721 as indicated for 24 h. γH2AX expression was detected in the cytoplasmic and nuclear extracts. Tubulin and TBP expression were used as loading controls. Original Western Blots of Figures S1 and S2 available in Figure S7.

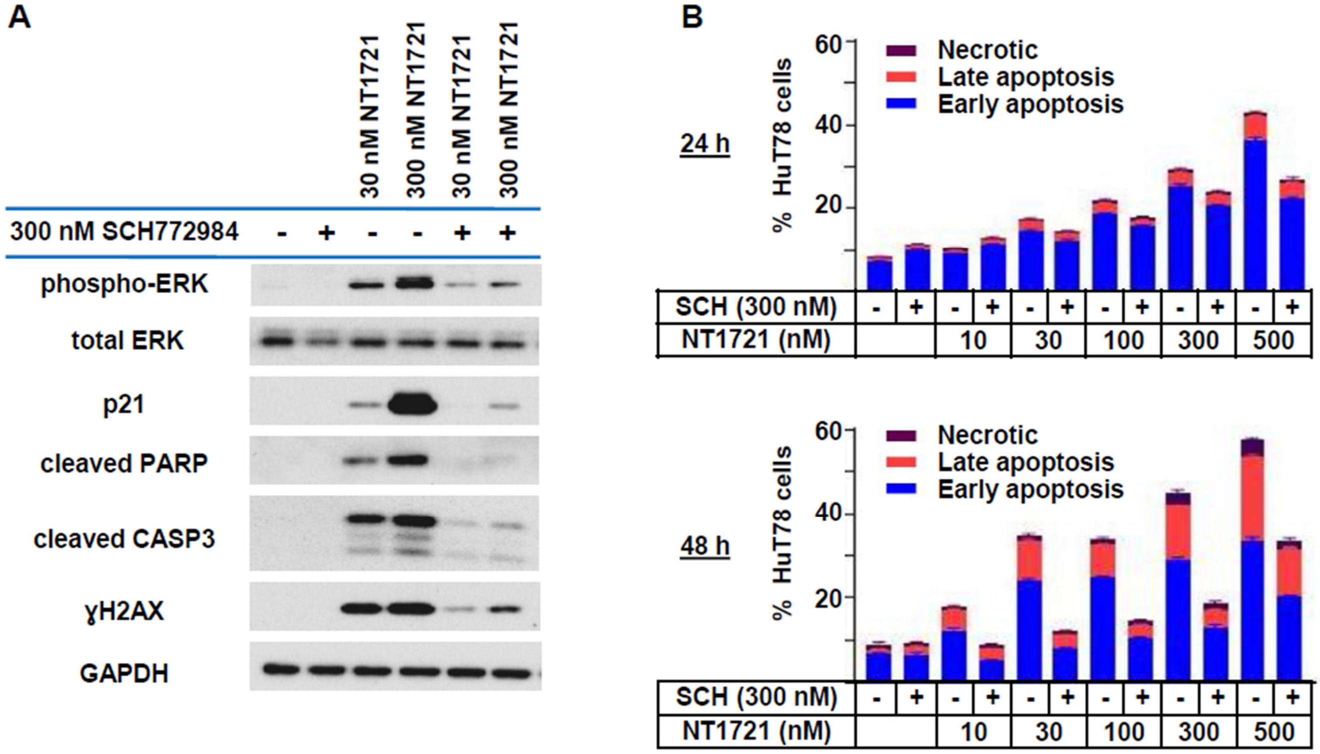

Figure S2. Combining NT1721 with an ERK inhibitor reduced apoptosis induction in HuT78 cells. (**A**) Protein expression in HuT78 cells treated with NT1721, the ERK inhibitor (SCH772984) or a combination. (**B**) Annexin V staining of HuT78

cells treated with NT1721, SCH772984 or combinations of both agents. Original Western Blots of Figures S1 and S2 available in Figure S7.

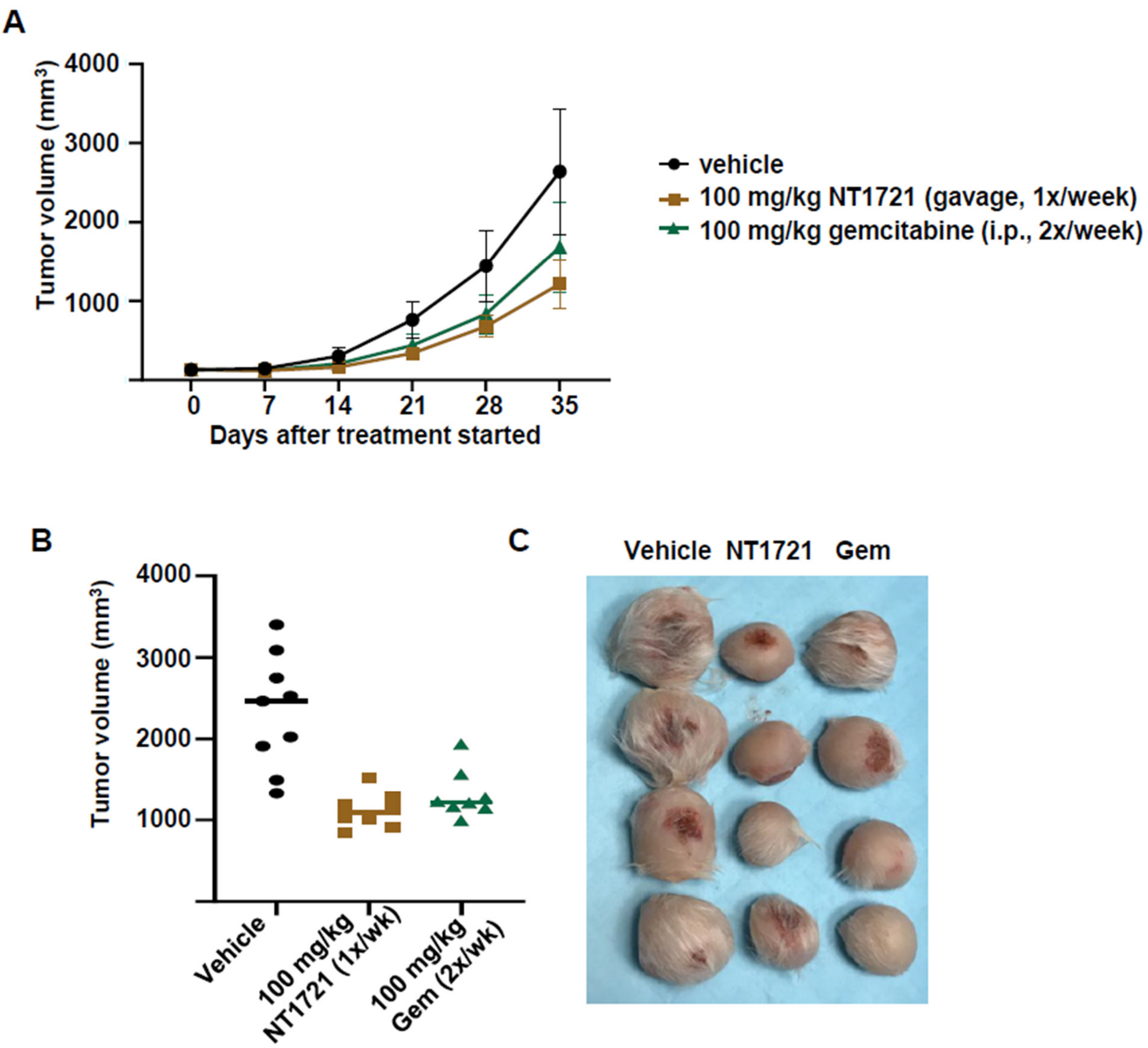

Figure S3. In vivo efficacies of NT171 (in DMSO-free formulation) and gemcitabine against HH tumors. The mice were treated with NT1721, gemcitabine or the vehicle controls as indicated. (**A**) Tumor volume. (**B**) Tumor weight. (**C**) Picture of representative tumors from treatment and control groups.

Figure 2A

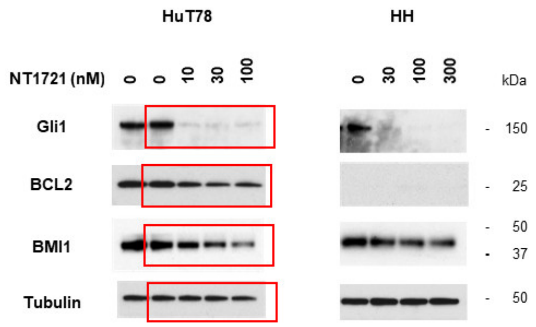

Figure 2C

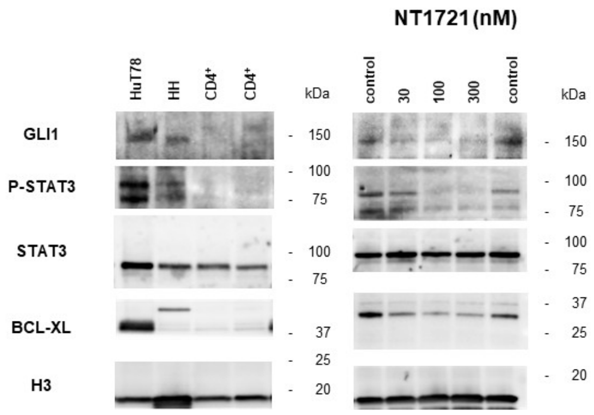

Figure S4. Original Western Blots of Figure 2A,C.

Figure 3B

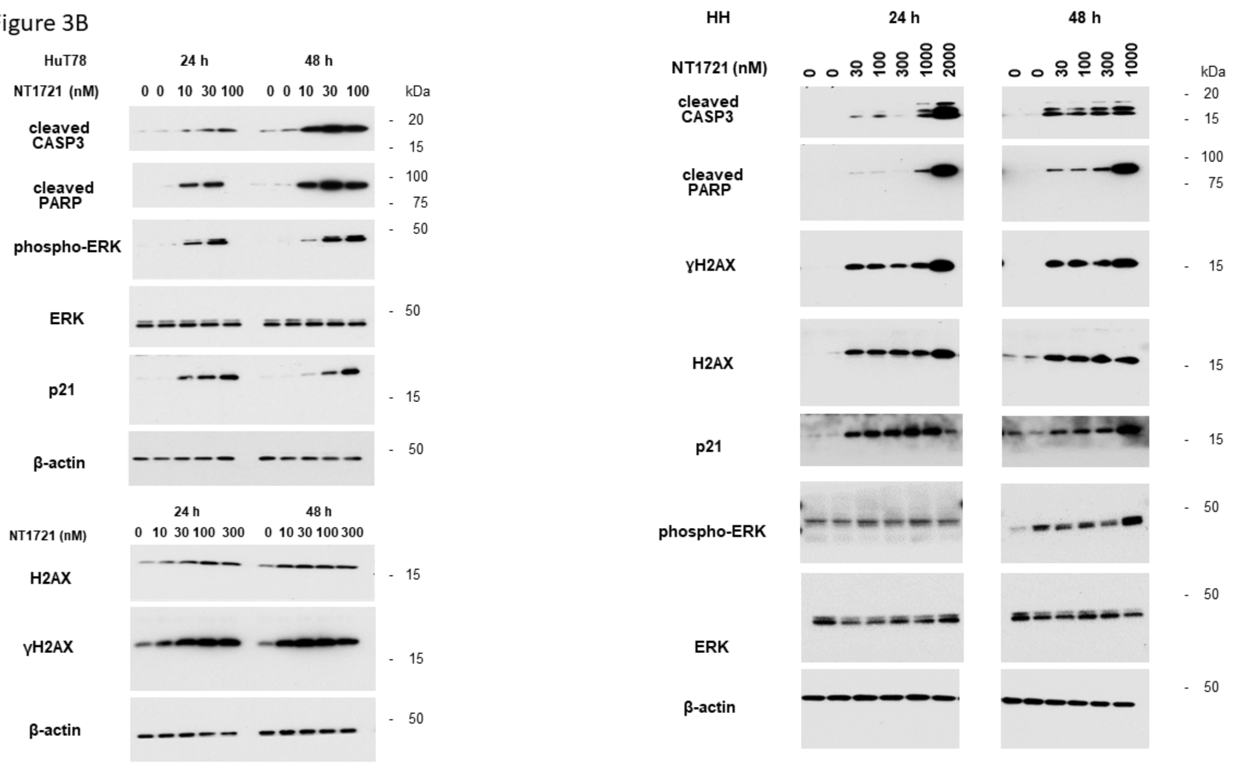

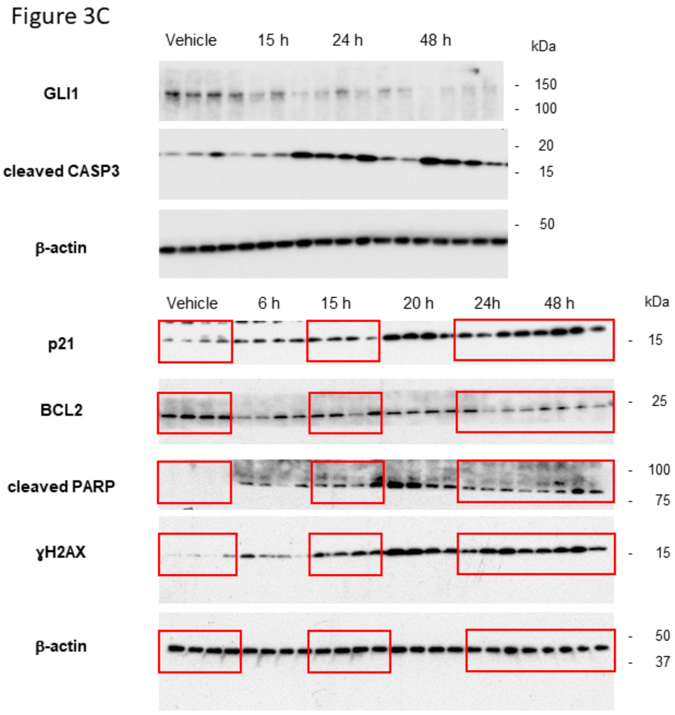

Figure S5. Original Western Blots of Figure 3B,C.

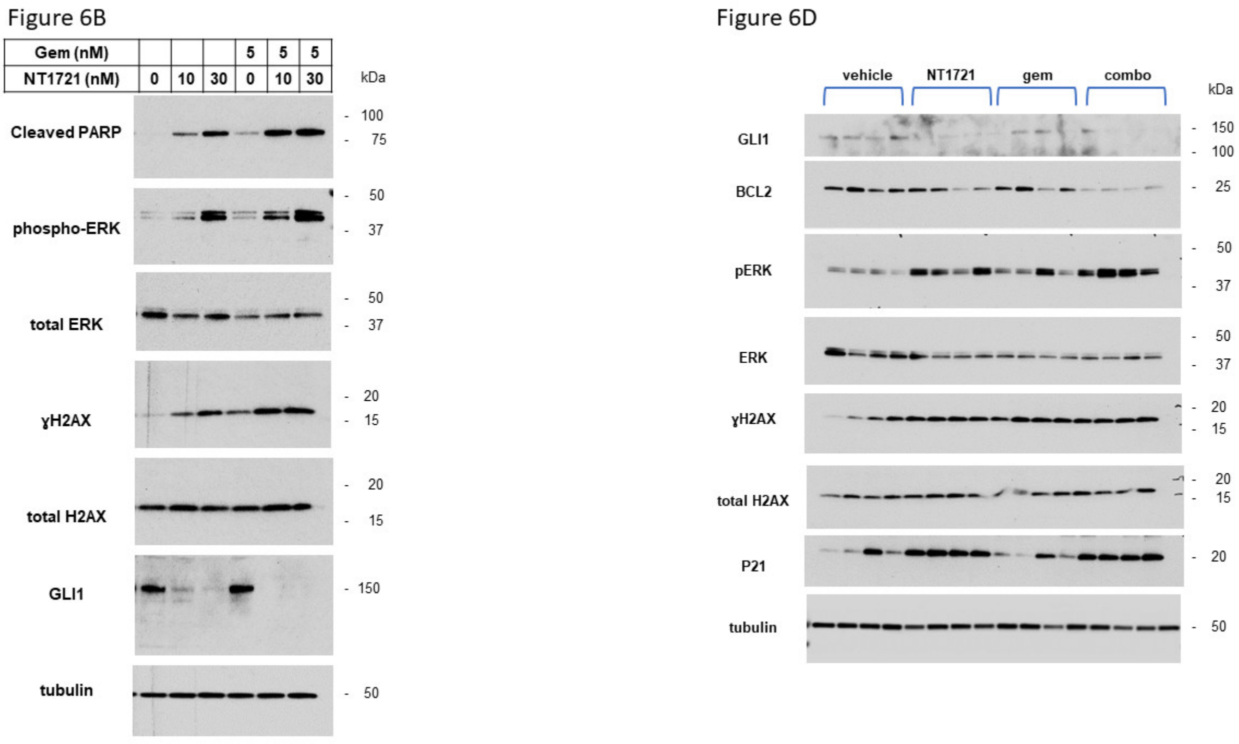

Figure S6. Original Western Blots of Figure 6B,D.

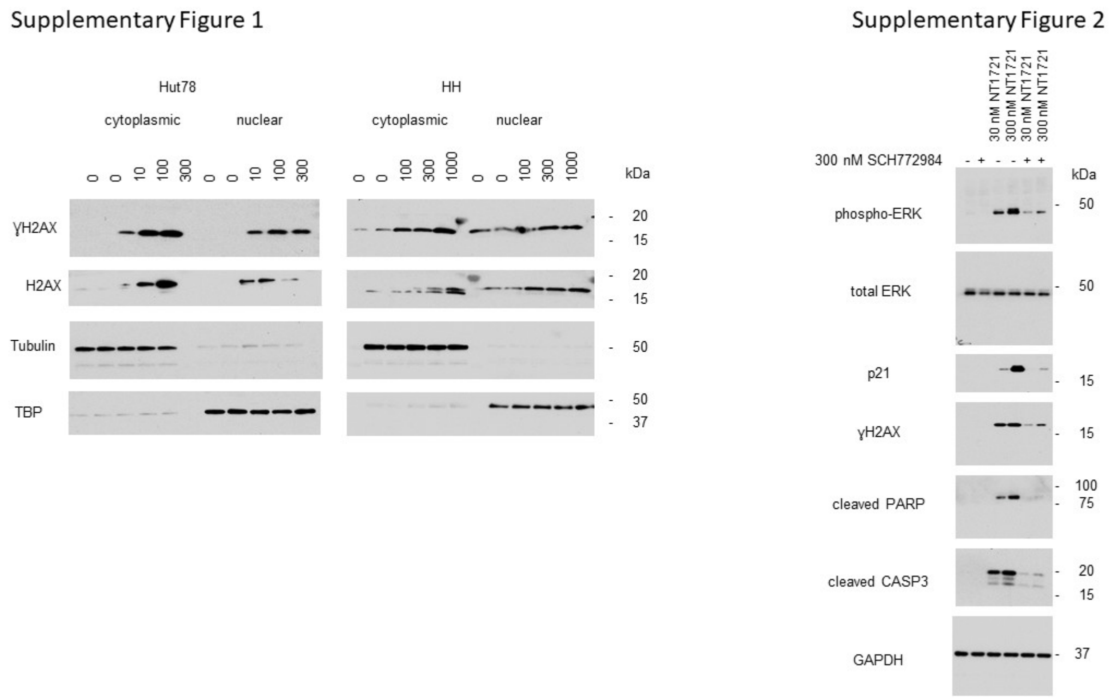

Figure S7. Original Western Blots of Figures S1 and S2.

Publisher's Note: MDPI stays neutral with regard to jurisdictional claims in published maps and institutional affiliations.

© 2021 by the authors. Licensee MDPI, Basel, Switzerland. This article is an open access article distributed under the terms and conditions of the Creative Commons Attribution (CC BY) license (http://creativecommons.org/licenses/by/4.0/).
